# The Chinese Hamster Ovary Cell-Based H9 HA Subunit Avian Influenza Vaccine Provides Complete Protection against the H9N2 Virus Challenge in Chickens

**DOI:** 10.3390/v16010163

**Published:** 2024-01-22

**Authors:** Shunfan Zhu, Zhenyu Nie, Ying Che, Jianhong Shu, Sufang Wu, Yulong He, Youqiang Wu, Hong Qian, Huapeng Feng, Qiang Zhang

**Affiliations:** 1 Department of Biopharmacy, College of Life Sciences and Medicine, Zhejiang Sci-Tech University, Hangzhou 310018, China; zhushunfan_2022@foxmail.com (S.Z.); nzy1997@icloud.com (Z.N.); shujianhong@zstu.edu.cn (J.S.); heyl79@zstu.edu.cn (Y.H.); 2 Zhejiang Novo Biotech Co., Ltd., Shaoxing 312366, China; y.che@novo-biotech.com (Y.C.); s.wu@novo-biotech.com (S.W.); y.wu@novo-biotech.com (Y.W.); h.qian@novo-biotech.com (H.Q.)

**Keywords:** H9N2 avian influenza virus, hemagglutinin, CHO cell, subunit vaccines, chicken

## Abstract

(1) Background: Avian influenza has attracted widespread attention because of its severe effect on the poultry industry and potential threat to human health. The H9N2 subtype of avian influenza viruses was the most prevalent in chickens, and there are several commercial vaccines available for the prevention of the H9N2 subtype of avian influenza viruses. However, due to the prompt antigenic drift and antigenic shift of influenza viruses, outbreaks of H9N2 viruses still continuously occur, so surveillance and vaccine updates for H9N2 subtype avian influenza viruses are particularly important. (2) Methods: In this study, we constructed a stable Chinese hamster ovary cell line (CHO) to express the H9 hemagglutinin (HA) protein of the major prevalent H9N2 strain A/chicken/Daye/DY0602/2017 with genetic engineering technology, and then a subunit H9 avian influenza vaccine was prepared using the purified HA protein with a water-in-oil adjuvant. (3) Results: The results showed that the HI antibodies significantly increased after vaccination with the H9 subunit vaccine in specific-pathogen-free (SPF) chickens with a dose–dependent potency of the immunized HA protein, and the 50 μg or more per dose HA protein could provide complete protection against the H9N2 virus challenge. (4) Conclusions: These results indicate that the CHO expression system could be a platform used to develop the subunit vaccine against H9 influenza viruses in chickens.

## 1. Introduction

Avian influenza virus (AIV) belongs to the family *Orthomyxoviridae*, genus influenza virus, and influenza A virus, and is also known as single and negative-stranded segmental RNA virus. It is divided into 18 subtypes (H1–H18), according to the antigenic difference of hemagglutinin (HA), and 11 subtypes (N1–N11), according to the difference of neuraminidase (NA) [1]. Influenza A viruses can be divided into highly pathogenic avian influenza viruses (HPAIVs) and low pathogenic viruses (LPAIVs), according to their pathogenicity in chickens. H9N2 belongs to the LPAIV, which induces highly contagious disease [2]. In 1994, H9N2 avian influenza first broke out in mainland China and spread rapidly across the country in just a few years. The percentage of chickens infected with the H9N2 subtype of avian influenza was up to 93.89% from 1996 to 2000 [3]. Despite large-scale vaccinations, surveillance in South China showed that the positive rate for H9N2 influenza viruses still accounted for 78.18% during 2013–2018, so H9N2 AIV remains one of the major AIV subtypes, in addition to H5N1 and H7N9 [4].

As an RNA virus, H9N2 is highly susceptible to antigenic drift and transfer and has not received sufficient attention and monitoring due to its low pathogenicity [5]. Summarizing the sporadic surveillance results, it is clear that along with large-scale infections, H9N2 AIV is breaking the species barrier, adding a range of hosts to the original birds and poultry, including pigs, dogs, minks, horses, rabbits, and bats [6,7,8,9,10]. In addition, the ability of H9N2 AIV to bind to mammalian upper respiratory tract cells is being gradually strengthened due to mutations in the HA protein, and human beings have also become hosts of H9N2 viruses [11]. The wide range of hosts and the large-scale spread of the virus have brought great harm to human production and life.

Related studies have reported that H9N2 avian influenza can cause respiratory symptoms in poultry, reduce egg production and fertilization rates, and cause secondary infections, posing a great threat to the poultry industry. In addition, the number of cases infected with H9N2 AIV in humans has been rising year by year. Since the first case was reported in Hong Kong in 1999, more than ninety-five cases have been reported to the World Health Organization (WHO), including one death [12,13]. It is worth noting that large-scale H9N2 infections in poultry provide an extensive gene pool for other subtypes of influenza viruses, and not only can H9N2 be extensively recombined to H1N1 and H3N2 [14,15,16] but some internal genes of H9N2 have been shown to enhance the pathogenicity of other subtypes of AIV, and the replacement of the polymerase acidic (PA) and nucleoprotein (NP) genes of H9N2 significantly enhances the pathogenicity of H5N1 in mice [17]. There is evidence that the recombinant H7N9 AIV from the 2013 outbreak derived its internal genes directly from H9N2 AIV [18]. Therefore, H9N2 is a major potential threat to the next influenza pandemic.

Similar to other viral diseases, vaccination remains the most effective strategy to control AIV [19]. Since the first inactivated H9N2 vaccine was approved in 1998, various types of monovalent and combined vaccines have been applied and have played an important role in preventing the spread of H9N2 AIV in China [20]. The inactivated vaccines are usually produced using chicken embryos [21] or MDCK cells [22]. This method of vaccine production has the advantage of easy storage and safety and is, therefore, very popular. However, antigenic protein loss during inactivation is a problem with the inactivated whole virus. Thus, a larger dose is usually administered, and an oil-based adjuvant is required to achieve expected immunity, which increases the cost of the vaccine and results in some side effects [23]. In addition, it is difficult to distinguish the infected animals and the vaccinated animals after we used the whole inactivated virus vaccines to immunize the poultry [24]. The proliferation of the influenza virus needs to use a large number of eggs, and the treatment of the used eggs also causes some environmental problems [25,26], so the development of new forms of vaccines is urgent.

In addition to inactivated vaccines, current new research directions include virus-like particles [27], recombinant vector vaccines [28], live attenuated vaccines [29], nucleic acid vaccines [30], and subunit vaccines [31]. The development of different types of vaccines provides new options for the control of H9N2 AIV. Virus-like particles are highly productive, but the purification of recombinant particles is complicated [32]. Recombinant vector vaccines and live attenuated vaccines have good immunity effects, but they need to maintain the viability of the virus, so the requirements for vaccine preservation and transportation are higher. Nucleic acid vaccines have a short development cycle, but the efficiency of in vivo expression is not high, and there is a risk of recombination with the host genome. Subunit vaccines have a high safety profile, but they require high-dose inoculation and have high production costs. The various forms of vaccines have demonstrated their respective advantages, among which subunit vaccines are highly expected due to their high safety but have not yet been effectively developed.

As the main surface antigen of the influenza virus, the HA protein is responsible for binding and entering the host cells [33], which can induce the production of neutralization antibodies and also stimulate the body to produce cellular immunity [34]. Genetically engineered vaccines based on HA protein have shown good immunogenicity and promising applications [35,36]. The expression systems currently used for HA protein production mainly include *Escherichia coli* [31], yeast [37], and insect cells [38]. These expression systems lack the ability to modify protein glycosylation as in mammalian cells, which play an important role in HA antigenicity. Therefore, there is an urgent need to develop a mammalian expression system for expressing HA proteins.

Chinese hamster ovary (CHO) cells are very popular in the production of subunit vaccines and protein drugs for human use [39]. Until now, the generation of a subunit vaccine for the H9N2 AIV using the CHO expression system is still undocumented. To explore this question in depth, we generated a CHO cell line stably expressing glycosylated HA protein to develop a novel subunit vaccine for H9N2 AIV using the recombinant plasmid, the pEE12.4-AIV-HA (H9). We further evaluated the immunogenicity and the efficacy of the CHO-based H9 HA subunit vaccine against virus challenges in chickens.

## 2. Materials and Methods

### 2.1. Cells and Viruses

CHO cells purchased from ATCC (Cat No. CBP60296) were maintained in Dulbecco’s Modified Eagle Medium (DMEM; Gibco, Cat No. 11965092) supplemented with 10% fetal bovine serum (FBS; Hyclone, Logan, UT, USA, Cat No. SH30066.03HI) and Penicillin-Streptomycin antibiotics.

A H9N2 AIV (A/chicken//Hebei/H9N2/2015) was proliferated in 10-day-old embryonated SPF chicken eggs for 72 h at 37 °C in Biosafety level-2 facilities. The virus titer was determined by detecting the 50% embryonic infectious dose (EID50) and was calculated using the formula derived from the Reed and Muench method [40].

### 2.2. Optimization and Synthesis of H9 HA Gene

The predominant circulating H9N2 strain A/chicken/Daye/DY0602/2017 (GenBank: MF794999) was selected. The HA sequence was used as a codon-optimized template, the optimized HA gene was synthesized by Nanjing Genscript Biotechnology Co., Ltd., (Nanjing, China), and the synthesized AIV- H9HA gene was cloned into pUC57 vector using Hind III and EcoR I enzyme digestion, termed pUC57-H9HA.

### 2.3. Construction of Recombinant Plasmids and Transfection

The HA gene and the pEE12.4 vector were treated with two restriction enzymes, Hind III and EcoR I, respectively, and then ligated together with T4 DNA ligase to obtain the recombinant plasmid, pEE12.4-HA. The plasmids were confirmed with no unwanted mutation using Sanger sequencing. Endotoxin-free plasmids were prepared for transfection, according to the manufacturer’s instructions (Thermo Fisher Scientific, Waltham, MA, USA, Cat No. A35892).

CHO cells were seeded into 6-well plates at 5 × 10^5^ cells/well. After 24 h culture and when the cells reached 60–80% confluence, the medium was removed, washed twice with PBS, and 800 μL of Opti-MEM was added. Dilute 2.5 μg of recombinant plasmid pEE12.4-HA and 7.5 μL of LTX transfection reagent with 125 μL of Opti-MEM, respectively, according to the manufacturer’s instructions, and then add 2.5 μL plus, mix mildly, and incubate for 5 min. Then, mix the plasmids and transfection reagent slowly and incubate them for 10 min at RT. Finally, add the mixture dropwise to CHO cells, gently shake the plate, and then change to DMEM medium with 10% FBS after 6 h and cultur the transfected cells for 48 h.

### 2.4. Screening and Generation of the Stable and H9-HA High-Expression Monoclonal Cell Line

CHO cells were plated into 6-well plates and treated with different concentrations of puromycin. The lowest puromycin concentration at which all cells died after 5 days was used for selection. The pEE12.4-HA-transfected cells were incubated with a complete medium containing an optimized concentration of puromycin 1 day after transfection. After all the cells in the negative control wells died, the supernatant of pEE12.4-HA-transfected cells was collected, and the HA protein containing the His tag was detected using sodium dodecyl sulfate–polyacrylamide gel electrophoresis (SDS-PAGE) and Western blotting. The positive cells were treated with trypsin and plated into a 96-well plate, 1 cell/well, and were cultured to obtain the monoclonal cell line, and then the cell line with the high-level expression was selected using Western blotting.

### 2.5. Expression of HA Proteins of H9N2 AIV

The transfected cell supernatant and monoclonal cell line supernatant were separated by SDS-PAGE and stained directly or transferred to a polyvinylidene fluoride (PVDF) membrane, respectively. The PVDF membranes were blocked overnight using PBS with 0.05% Tween 20 (PBST) with 5% skim milk powder and washing with PBST and were then incubated for 2 h with a 1:1000 dilution of anti-His tag mouse monoclonal antibody (HRP) (Beyotime, Shanghai, China, Cat No. AF2879). After washing with PBST, the expression of HA protein was detected using an Omni-ECL™Femto Light Chemiluminescence Kit (Shanghai Epizyme Biomedical Technology Co., Ltd., Shanghai, China, Cat No. SQ201).

### 2.6. Protein Purification and Vaccine Preparation

The cell supernatant after centrifugation was added with a protease inhibitor and passed through a 0.8 μm filter. The protein was purified using a nickel column from the supernatant. At first, we treated the nickel column, added the proper volume of nickel agarose, and then added ddH_2_O to 2/3 of the column, inverted gently to mix, opened the bottom cover to flow out the liquid, and repeated three times. Next, add 5 times the column volume of His Buffer B solution (weight 2.42 g Tris, 11.7 g NaCl, and 34 g imidazole, add 100 mL glycerol, dissolve in 900 mL ddH_2_O, adjust the pH to 8.0 with concentrated HCl, add ddH_2_O to make the volume 1 L, and store at room temperature until use), incubate on ice for 10 min at 60 rpm, and flow the buffer out from the bottom. Then, add 5 times the column volume of His Buffer A solution (weight 2.42 g Tris, 11.7 g NaCl, 1.7 g imidazole, add 100 mL glycerol, dissolve in 900 mL ddH_2_O, adjust the pH to 8.0 with concentrated HCl, add ddH_2_O to make the volume 1 L, and store at room temperature until use), incubate on ice for 30 min at 60 rpm, and flow the buffer out; slowly add the cell supernatants and incubate them on ice for 4 h at 60 rpm, add 5 times the volume of His Buffer A solution, incubate on ice for 15 min at 60 rpm, collect the flow-through fluid, and repeat 3 times for washing, and then add 5 times the volume of His Buffer B solution, incubate on ice for 15 min at 60 rpm for elution, collect the flow-through fluid, and repeat twice to obtain the purified protein (Solarbio, Beijing, China). The purified protein was dialyzed in PBS, and the protein concentration was determined using the bicinchoninic acid (BCA) method.

### 2.7. Immunization and Protection

The HA protein expressed in CHO cells in PBS was added with a sterilized Marcol-52 (ExxonMobil, Houston, TX, USA) adjuvant, emulsified, and prepared as a subunit vaccine with 200 μg/mL protein concentration. Four-week-old specific-pathogen-free (SPF) chickens were purchased from Beijing Boehringer Ingelheim Vital Biotechnology Co., Ltd., Beijing, China. After 1 week of adaption, 35 SPF chickens were randomly divided into 5 groups, with 7 chickens/group. Among them, 4 groups were immunized with 12 μg/dose, 25 μg/dose, 50 μg/dose, and 100 μg/dose, respectively, and a group was injected with PBS as a negative control. The vaccine was administered intramuscularly through the chest, and the immunization was performed twice at a two-week interval; blood was collected on days 14 and 21 after the second immunization, and the sera were isolated.

On day 21, after the second immunization, the H9N2 virus was diluted with PBS, and we inoculated the chickens with 0.2 mL/chicken (about 2.5 × 10^7^ EID_50_) via intravenous injection. Oropharynx swabs and cloacal swabs were collected on day 5 after the challenge and suspended in 1 mL of PBS containing antibiotics (10,000 U/mL of penicillin and 10 mg/mL of streptomycin). After centrifugation, 200 μL of the supernatant was inoculated with 10-day-old chicken embryos, and after 48 h, the allantoic fluid was collected and analyzed using an HA assay.

### 2.8. HI Assay for Determining the Antibody Titers

The HI antibodies were detected as described previously [41,42]. Briefly, a 25 µL sample of sera was continuously 2-fold diluted in a 96-well plate in PBS, followed by the addition of 25 µL of 4 units of an AIV antigen (Harbin National Engineering Research Center of Veterinary Biologics Corporation, Harbin, China) and incubated for 20 min at RT for antigen–antibody binding. Then, 25 µL of 1% chicken red blood cells were added and incubated at RT for 20 min. The hemagglutination inhibition antibody titer is defined as the reciprocal of the maximum dilution that inhibits the agglutination of red blood cells after 20 min.

## 3. Results

### 3.1. Construction of Recombinant Plasmids

**Figure 1:**
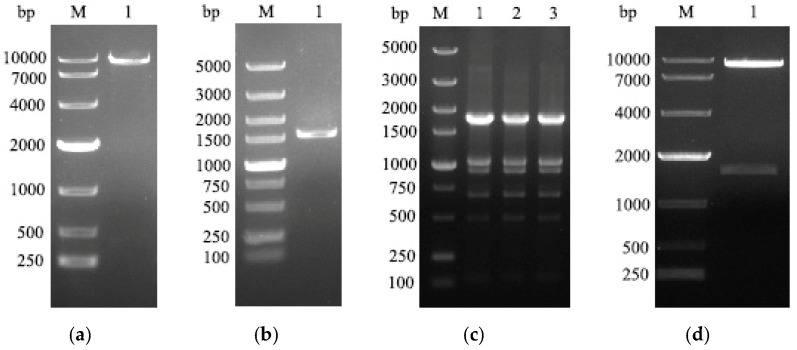
Construction of recombinant plasmids: (**a**) a fragment of the pEE12.4 vector after enzymatic cleavage was approximately 8746 bp; (**b**) the soluble fragment of the HA gene after enzymatic gel recovery was about 1551 bp; (**c**) after transformation of the recombinant plasmid into DH5α, we picked three recombinant bacterial clones, and the selected recombinants were identified by PCR in bacterial broth and were approximately 1788 bp; (**d**) the recombinant plasmid was verified by double restrict enzyme (H*ind* III and E*coR* I) digestion with approximately 8746 bp and 1551 bp.

The vector, pEE12.4, and the target gene, HA, were digested with double restriction endonucleases, H*ind* III and E*coR* I, respectively, and the correct gene fragments were recovered at approximately 8746 bp and 1551 bp (Figure 1a,b). After transforming the recombinant plasmid into DH5α component cells, the spots of bacteria were selected for PCR verification, and the PCR product was about 1788 bp, which verified the construction of the recombinant plasmid (Figure 1c). Then, the plasmid was extracted for double digestion verification, and two correct bands were obtained, about 1551 bp and 8746 bp (Figure 1d), and the recombinant plasmid was sequenced to confirm no unwanted mutation. These results indicate that the recombinant plasmid, pEE12.4-HA, has been successfully constructed.

### 3.2. Expression of HA Protein in CHO Cells

**Figure 2:**
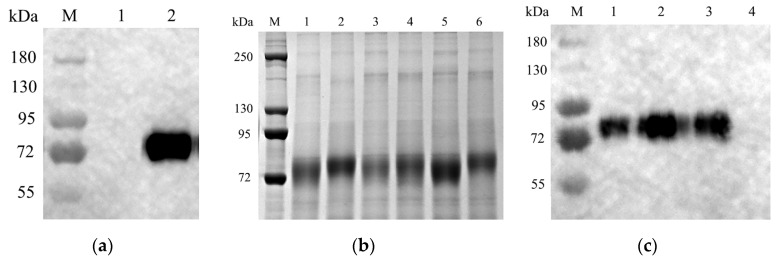
Expression of HA protein in CHO cells: (**a**) Western blotting assay of supernatant from recombinant plasmid transfected CHO cells for 96 h. M: protein markers, 1: negative control, 2: the supernatant sample; (**b**) SDS-PAGE assay of the selected CHO monoclonal cell line supernatant. M: protein markers, 1–6: different samples collected from monoclonal CHO cell lines; (**c**) Western blotting assay of monoclonal cell line supernatant. M: protein marker, 1–3: different monoclonal cell lines, 4: negative control.

The endotoxin-free recombinant plasmid was transfected into CHO cells, according to the manufacturer’s instructions. After transfection for 96 h, the expression of HA protein in the transfected supernatant was detected by Western blotting. The molecular size of the expressed HA protein was presented as approximately 85 kDa. This result demonstrated that the H9 HA protein was expressed in CHO cells (Figure 2a). Transfected CHO cells were selected with puromycin, and monoclonal cell lines were obtained by the limited dilution method. After the monoclonal cells in the 96-well plate were cultured to 80–90% confluence, the supernatant of each well was collected for SDS-PAGE and Western blotting detection (Figure 2b,c). These results indicated that we have obtained the monoclonal CHO cell lines with a high expression of HA protein.

### 3.3. The H9N2 Virus-Specific Antibodies Were Induced after Being Vaccinated with the H9 Subunit Vaccine Produced in CHO Cells

**Figure 3:**
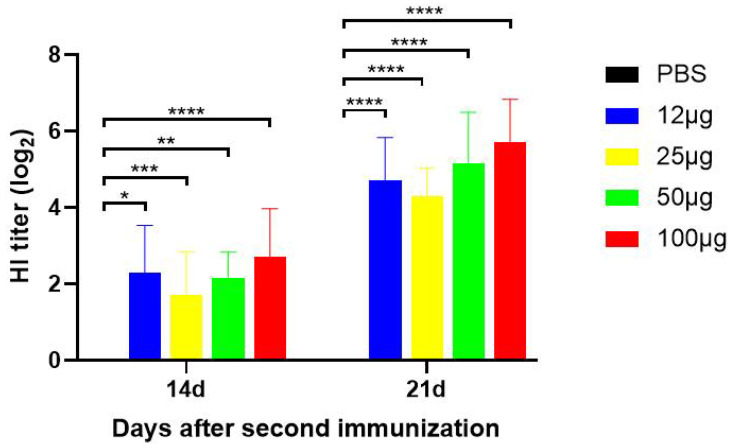
Detection of HI antibody titers in the SPF chicken sera. All results are presented as mean ± SD (standard deviation). Two-way ANOVA was used for significant analysis. * *p* < 0.05, ** *p* < 0.01, *** *p* < 0.001, **** *p* < 0.0001.

The SPF chickens were immunized with the H9 HA subunit vaccine twice at a two-week interval. As shown in Figure 3, on day 14 and day 21 after the second immunization, in addition to the PBS group, the lowest dose of immunization (12 μg HA) also induced detectable HI antibodies, and the HI antibody titers were dose–dependent. The group with 100 µg/dose produced the highest antibody titers. In this group, the mean HI antibody titer could reach up to 5.71 log2 on day 21 after the second immunization. These results suggest that the HA protein produced by CHO cells possesses good immunogenicity and could induce the expected humoral immune response.

### 3.4. The H9 HA Vaccine Provides Complete Protection from the H9N2 AIV Virus Challenge

After three weeks of the second immunization, the immunized chickens were infected with 2.5 × 10^7^ EID_50_ H9N2 virus with intravenous injection. Orpharyngeal swabs and cloacal swabs were collected on day 5 after the challenge. The swabs were placed into sterile PBS, and then the virus titers were determined in 10-day-old SPF embryonated chicken eggs. The results showed that no viruses were detectable in Orpharyngealswabs and cloacal swabs in all immunization groups in the 50 µg per dose and 100 µg per dose group (Table 1). A total of 50 μg per dose could provide complete protection against the H9N2 virus challenge.

## 4. Discussion

Avian influenza was first discovered in Italy in 1878 and is one of the major infectious diseases to affect poultry production and human health [43]. H9N2 AIV has attracted widespread attention as one of the most predominant subtypes [44]. Although the risk posed by H9N2 AIV has been decreasing, along with large-scale vaccination and the escalation of prevention policies, there is still no effective means to completely interdict the spread of H9N2 AIV [19]. On the other hand, the chickens that were infected with H9N2 were easier to be infected by other pathogens, such as bacteria; in this case, the mixed infection caused more severe disease in poultry. The prevention and control of H9N2 AIV infection plays a crucial role for poultry, especially in spring and winter.

As an excellent protein expression platform, the CHO system has been validated in the production of various recombinant protein drugs and monoclonal antibodies. In a study by Lin et al. comparing the immunogenicity of H5N1 AIV HA proteins produced by mammalian and insect cell expression systems, it was demonstrated that HA proteins produced by the CHO expression system induced the production of higher levels of neutralizing antibody titers and hemagglutination-inhibiting antibodies [45]. In addition, it was found that H7N9 AIV HA proteins produced by CHO cells in combination with the nanoemulsion PELC/CpG induced the production of high titers of neutralizing antibodies and provided protection against live virus challenge [46]. The feasibility of utilizing the CHO expression system for the production of AIV subunit vaccines is now well-established. CHO cells have fewer inner proteins and can express some target protein in the supernatant; therefore, these advantages of CHO cells make the expressed protein easier than other expression systems. A CHO-based RBD COVID-19 vaccine was approved in China during the SARS-CoV-2 pandemic [47].

In this study, the HA gene was stably integrated into the genome of CHO cells using genetic recombinant technology, and CHO cell lines that could stably express HA protein at high levels were obtained by antibiotic selection. In addition, the HA protein expressed by CHO cells was about 85 kDa (Figure 2), which was much larger than the expected molecular weight of 60 kDa [48], demonstrating the ability of the CHO cell line to express glycosylated modifications of the exogenous protein. The purified HA protein was emulsified with an adjuvant to develop a subunit vaccine against H9N2 AIV. The results of the HI antibody potency test in the serum of SPF chickens showed that the specific antibody level induced by HA protein was dose–dependent on the protein content, and the highest HI antibody level could reach 5.71 log2 on the 21st day after boost immunization, which could provide protection for SPF chickens (Figure 3). Detection of viral shedding on the 5th day after challenge showed that all immunized groups as low as 12 µg were effective in blocking cloacal virus shedding, and vaccine groups with doses of 50 µg and above were effective in blocking oral virus shedding (Table 1). Therefore, oral virus shedding detection is more important than cloacal virus shedding.

Glycosylation is a common post-translational modification and is important for protein biology. HA protein is the main antigen of the AIV. N-glycosylation of HA plays a key role in receptor binding, immune response, and pathogenicity [49]. Glycosylation in the stalk region contributes to the proper folding and trimerization and is highly conserved among different viral strains. On the other hand, glycosylation in the head domain is mainly involved in antigenicity change, and different subtypes of AIVs possess different distribution patterns. For example, two to six glycosylation sites are located in the HA head domain of H3N2, while H7N9 has only one glycosylation site [50]. Furthermore, a different glycan pattern of the same protein was presented and derived from different mammalian cells [51,52]. In this study, we found that H9 HA expressed in the CHO cells was bigger than expected (Figure 2a). H9 HA usually has six potential glycosylation sites in HA1 (11–13 NST, 123–125 NVS, 200–202 NRT/I/N, 280–282 NTT, 287–289 NVS, 295–297 NCS) and two glycosylation sites in HA2 (154–156 NGT, 213–215 NGS) [53]. Therefore, the bigger HA protein was caused by glycosylation in CHO cells since the H9 HA expressed in this study included at least seven potential above glycosylation sites.

Yield is one important factor in the cost of vaccines. In this study, H9 HA reached 1 g or more per liter, which is higher than the yield of the HA in the baculovirus system, which is 30 mg/L [54]. Compared to the whole inactivated H9 AIV vaccine, the CHO-based H9 subunit vaccine has several advantages. The first benefit is that H9 stable expression CHO cells produce H9 HA protein continuously, and it is easy to perform large-scale production. The second advantage is that the production process did not need to amplify the large number of H9N2 AIVs and did not have any biosafety threat; it also did not need to use large numbers of embryo eggs to reduce the effect on the environment of the used eggs. Another disadvantage of the whole inactivated H9 AIV is that this type of vaccine could not distinguish between the naturally infected animals and vaccinated animals. A CHO-based H9 HA vaccine only induces the specific antibodies targeted to the HA, so it is easy to distinguish the infected and vaccinated animals using the specific antibody targeting other proteins of the AIVs [55].

Currently, the water-in-oil adjuvant is the main adjuvant for making the AIV vaccines, including the H9N2 virus vaccine used in chickens. The next generation of poultry vaccines should consider using the aqueous adjuvant to improve the meat quality around the injection sites. Additionally, since H9N2-infected cases occurred occasionally in humans and other mammals, a CHO-based H9 HA vaccine could be potentially developed for use for humans or other mammals matched with proper adjuvants in the future, such as MF59 and AS03 [56,57].

In conclusion, our study showed that the HA subunit vaccine produced based on the CHO expression system induced an increase in the HI antibody level of SPF chickens and effectively responded to the attack of the H9N2 virus and inhibited the spread of the virus. The CHO expression system is a good platform for the production of a subunit vaccine against H9N2 AIV.

## 5. Patents

This work has been filed for a patent application by Zhejiang Novo Biotech Co., Ltd. (Shaoxing, China). 

## Figures and Tables

**Table 1:** Virus shedding from the immunized chicken after the H9N2 virus challenge.

	PBS	12 μg	25 μg	50 μg	100 μg
Orpharyngeal swabs	7/7	4/7	6/7	0/7	0/7
Cloacal swabs	4/7	0/7	0/7	0/7	0/7

Each swab corresponds to one SPF chicken, and 1 mL of swab dilution is inoculated into five SPF chicken embryos. The HA titer of the chicken embryo’s allantoic fluid was detected after 48 h. As long as the HA test of the allantoic fluid of one chicken embryo was positive, the virus was determined to be isolated positively.

## Data Availability

Data are contained within the article.
